# On the Meaning of De‐Excitations in Time‐Dependent Density Functional Theory Computations

**DOI:** 10.1002/jcc.70072

**Published:** 2025-03-25

**Authors:** Felix Plasser

**Affiliations:** ^1^ Department of Chemistry Loughborough University Loughborough UK

**Keywords:** excited states, quantum chemistry, time‐dependent density functional theory, unpaired electrons

## Abstract

De‐excitations play a prominent role within the mathematical formalism of time‐dependent density functional theory (TDDFT) and other excited‐state response methods. However, their physical meaning remains largely unexplored and poorly understood. It is the purpose of this work to shed new light on this issue. The main thesis developed here is that de‐excitations are not a peculiarity of TDDFT but that they are a more fundamental property of the underlying wave functions reflecting how electrons are excited between partially occupied orbitals. The paraquinodimethane (pQDM) molecule is chosen as a convenient model system whose open‐shell character can be modulated via twisting of its methylene groups. Using the one‐electron transition density matrix as a rigorous basis for our analysis, we highlight qualitative and quantitative parallels in the way that de‐excitations are reflected in multireference wave function and TDDFT computations. As a physically observable consequence, we highlight a lowering of the transition dipole moment that derives from destructive interference between the excitation and de‐excitation contributions. In summary, we hope that this work will shed new light on formal and practical aspects regarding the application of TDDFT to excited‐state computations, especially of diradicaloid systems.

## Graphical abstract

De‐excitations play a central role in the mathematical formalism of time‐dependent density functional theory, but their physical meaning has not been studied in detail. This work sheds new light onto this issue by showing that de‐excitations arise naturally also in wave function based theories where they represent ground‐state correlation.

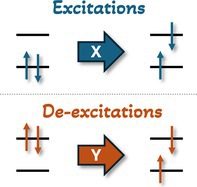

## 1 Introduction

De‐excitations play a prominent role in the mathematical formalism of time‐dependent density functional theory (TDDFT) [1, 2, 3] and other excited‐state response methods such as the second‐order polarization propagator (SOPPA) [4]. However, their physical meaning has remained largely unexplored. De‐excitations are often brushed aside as seemingly nonphysical or insignificant without further discussion. Work in this area has largely been constrained to immediate practical aspects, for example, the numerical stability of the Tamm–Dancoff approximation (TDA) [3, 5, 6] and the performance of full TDDFT for exciton binding energies [7]. Some more specific questions are concerned with how to interpret and analyze computations including de‐excitations [8] and how the de‐excitation contributions affect spin expectation values [9]. On a more formal level, it has been noted that full TDDFT is gauge invariant and satisfies certain sum rules whereas this is not the case for the TDA [2, 3, 10]. However, some, arguably more fundamental, questions have remained unanswered, in particular regarding the physical meaning of de‐excitations and whether they exist in wave function‐based computations.

It is the purpose of this work to shed light on the above questions. The main issue to be investigated is whether de‐excitations are indeed a physical phenomenon that exists independently of TDDFT and whether they can be understood on a more general method‐independent footing. Specifically, we will develop the idea that de‐excitations are not so much a characterization of the excited state as such, but that they are a reflection of unpaired electrons in the ground state. There exists a long tradition of quantifying unpaired electrons in the electronic ground state [11, 12, 13, 14, 15, 16, 17] and this work will build on these ideas, highlighting that the de‐excitation amplitudes can be seen as a measure for ground‐state unpaired electrons in their own right.

Aside from the fundamental question of what de‐excitations are, this work will also investigate the question of how TDDFT performs for open‐shell and diradicaloid systems. There has been a surge in the development of luminescent diradicals in recent years [18, 19, 20, 21], and there is a need for computational protocols for describing such systems effectively. Indeed, TDDFT based on a closed‐shell reference has the attractive property of providing spin‐adapted states in a very computationally efficient manner, and it is interesting to explore how far it can be applied when diradical character increases. While the question of how standard TDDFT, along with its broken‐symmetry and spin–flip variants, performs in the case of quasi‐degenerate and diradicaloid ground states has certainly been discussed before [22, 23, 24, 25], we will show how new insight can be gained through the lens of de‐excitations.

**FIGURE 1:**
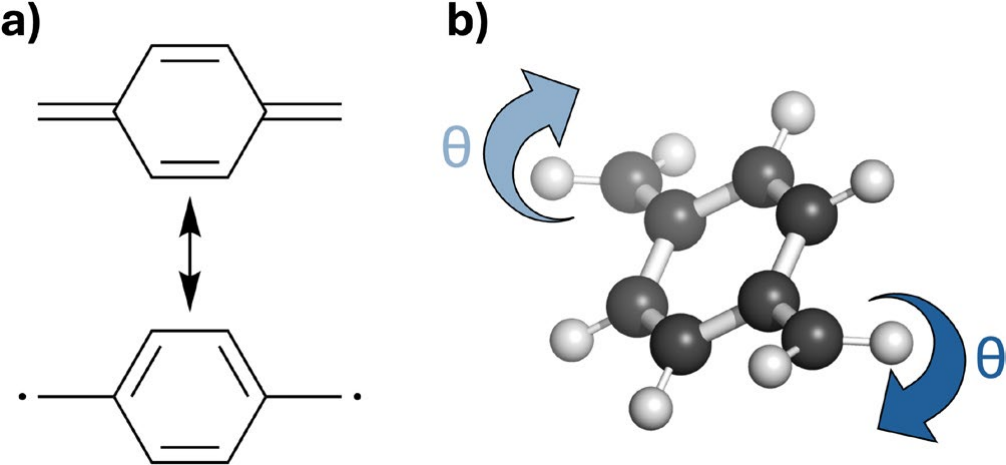
The pQDM molecule analyzed in this work: (a) depiction of relevant resonance structures, (b) illustration of the torsion angle (θ) relating to simultaneous twisting around the CH_2_ groups.

The molecule investigated here is paraquinodimethane (pQDM), illustrated in Figure 1. pQDM and its derivatives have been studied widely as molecules with tunable diradical character [26, 27, 28]. As shown in Figure 1a it can be drawn using a closed‐shell nonaromatic resonance structure or a diradical resonance structure with an aromatic sextet. This gain in aromaticity upon forming a diradical explains its partial diradical character in the ground state as well as a rather low‐lying first triplet state. In the present context pQDM serves as a convenient model system, which can be tuned from modest diradical character at its planar geometry to enhanced diradical character when its terminal CH_2_ groups are twisted. As illustrated in Figure 1b, we will twist the CH_2_ groups simultaneously along an angle denoted θ, always keeping both CH_2_ groups in the same plane. Here, increased twisting is intended to enhance diradical character. TDDFT and TDA computations will be performed and the results compared to high‐level multireference computations. We will investigate energies, transition dipole moments, as well as more intricate details of the transition density matrices involved.

Within this work, we start with a detailed Methods section explaining the specifics of TDDFT as well as general wave function analysis protocols. Subsequently, we present results on the pQDM molecule, starting with its planar geometry, providing a detailed comparison between TDDFT, TDA, and complete active space perturbation theory (CASPT2). We proceed by investigating how energies and wave functions change upon twisting of the CH_2_ groups. Finally, a short discussion of the implications of the main results is presented.

## 2 Methods

This section presents the underlying methods in some detail, starting with a discussion of the main TDDFT equations, pointing out how de‐excitations arise. This is followed by an explanation of how, using our general wave function analysis framework, de‐excitations can be defined in a method‐independent wave function‐based picture. After that, the connection between this wave function analysis framework and TDDFT is drawn. Subsequently, the main underlying physics and the expected results are analyzed using a diradical model system. Finally, computational details are presented.

### 2.1 TDDFT and De‐Excitations

The starting point of the discussion is the common random‐phase‐approximation‐like formulation [2, 29] of linear‐response (LR) TDDFT within the Kohn–Sham formalism, given as the following generalized matrix eigenvalue problem (assuming real valued matrices and vectors for simplicity)

(1) $$\left(\begin{array}{cc} A & B\\ B & A \end{array}\right)\left(\begin{array}{c} X\\ Y \end{array}\right)=\omega \left(\begin{array}{cc} 1 & 0\\ 0 & -1 \end{array}\right)\left(\begin{array}{c} X\\ Y \end{array}\right).$$

Here, the matrices A and B are the orbital Hessians; the vectors X and Y define the response function. For a hybrid functional (with constant admixture of nonlocal exchange $c_{\mathrm{HF}}$) and using the adiabatic approximation, the matrices A and B are defined as

(2) $$A_{\mathrm{ia},\mathrm{jb}}=\left(\mathrm{ia}|\mathrm{jb}\right)+\left(1-c_{\mathrm{HF}}\right)\left(\mathrm{ia}|f_{\mathrm{xc}}|\mathrm{jb}\right)-c_{\mathrm{HF}}\left(\mathrm{ij}|\mathrm{ab}\right)+\delta_{\mathrm{ij}}\delta_{\mathrm{ab}}\left(\epsilon_{a}-\epsilon_{i}\right)$$

(3) $$B_{\mathrm{ia},\mathrm{jb}}=\left(\mathrm{ia}|\mathrm{bj}\right)+\left(1-c_{\mathrm{HF}}\right)\left(\mathrm{ia}|f_{\mathrm{xc}}|\mathrm{bj}\right)-c_{\mathrm{HF}}\left(\mathrm{ib}|\mathrm{aj}\right)$$

where $\epsilon_{i}$ is an orbital energy, $\left(\mathrm{ia}|\mathrm{jb}\right)$ is a two‐electron repulsion integral written in Mulliken charge cloud notation, and $\left(\mathrm{ia}|f_{\mathrm{xc}}|\mathrm{jb}\right)$ is a two‐electron integral involving the response of the exchange‐correlation potential. Here and below, ij/ab/pq are used to represent occupied/virtual/general orbital indices, respectively.

The meaning of X and Y can be understood within an equation‐of‐motion formalism [9]. Here, the operator projecting the ground state $\Psi_{0}$ onto the *I*‐th excited state $\Psi_{I}$ is constructed using the X and Y vectors as

(4) $$\left|\Psi_{I}\right\rangle \left\langle \Psi_{0}\right|=\sum_{\mathrm{ia}}\left(X_{\mathrm{ia}}{\hat{a}}_{a}^{†}{\hat{a}}_{i}+Y_{\mathrm{ia}}{\hat{a}}_{i}^{†}{\hat{a}}_{a}\right)$$

where ${\hat{a}}_{a}^{†}$ and ${\hat{a}}_{i}$ are the creation and annihilation operators, respectively. Within the first part of this expression, electrons are promoted from occupied to virtual orbitals following the $X_{\mathrm{ia}}$ amplitudes, whereas they are “demoted” from virtual to occupied orbitals following the $Y_{\mathrm{ia}}$ amplitudes. These processes are often denoted excitations and de‐excitations determining the content of this work. To interpret Equation (4) in some more detail, it is helpful to project it onto $\left|\Psi_{0}\right\rangle$

(5) $$\left|\Psi_{I}\right\rangle =\sum_{\mathrm{ia}}\left(X_{\mathrm{ia}}{\hat{a}}_{a}^{†}{\hat{a}}_{i}+Y_{\mathrm{ia}}{\hat{a}}_{i}^{†}{\hat{a}}_{a}\right)\left|\Psi_{0}\right\rangle$$

This shows that the excited state can be constructed by excitations and de‐excitations acting on the ground state. The second, de‐excitation, part of this equation would necessarily vanish if $\Psi_{0}$ is a single determinant with orbitals i,j,… occupied and orbitals a,b,… unoccupied. Thus, de‐excitations are only pertinent if the ground state $\Psi_{0}$ is of multideterminantal character. Alternatively [30], one may regard the “killer condition” by applying the adjoint of the operator in Equation (4) on the ground state

(6) $$\left|\Psi_{0}\right\rangle \left\langle \Psi_{I}|\Psi_{0}\right\rangle =\sum_{\mathrm{ia}}\left(X_{\mathrm{ia}}{\hat{a}}_{i}^{†}{\hat{a}}_{a}+Y_{\mathrm{ia}}{\hat{a}}_{a}^{†}{\hat{a}}_{i}\right)\left|\Psi_{0}\right\rangle =0.$$

For non‐vanishing Y this can, again, only be fulfilled if the ground state includes contributions from excited determinants.

It is a curious property of Equation (1) that for every solution $\left(X,Y\right)$ with an eigenvalue ω, swapping the vectors X and Y will also produce a solution, but this time with the eigenvalue −ω [9]. This can be seen, for example, by viewing Equation (1) in a condensed form and then swapping the rows as well as extracting the minus sign on the right‐hand side

(7) $$\left(\begin{array}{c} \mathrm{AX}+\mathrm{BY}\\ \mathrm{BX}+\mathrm{AY} \end{array}\right)=\omega \left(\begin{array}{c} X\\ -Y \end{array}\right)\Leftrightarrow \left(\begin{array}{c} \mathrm{AY}+\mathrm{BX}\\ \mathrm{BY}+\mathrm{AX} \end{array}\right)=-\omega \left(\begin{array}{c} Y\\ -X \end{array}\right)$$

The associated pairs of solutions are termed excitations and de‐excitations. Note that the terms “excitation” and “de‐excitation” are, thus, used to describe the overall solutions as well as the individual orbital contributions. However, there is a clear connection: a positive‐energy solution is usually one with predominant orbital excitations, and a negative‐energy solution one with predominant orbital de‐excitations (except potentially in cases where excited states are near‐degenerate to the ground state). In the following, we will be concerned with the admixture of orbital de‐excitations into the excitation solutions.

Left‐multiplying Equation (1) by $\left(X^{T}\;Y^{T}\right)$ yields:
(8)

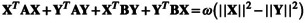




This suggests the commonly employed normalization condition for excitation solutions

(9) $$||X|{|}^{2}-||Y|{|}^{2}=1$$

which implies that the excitation energy can be read directly from the left‐hand side of Equation (8).

As a simplification to full TDDFT, one often applies the TDA in practical calculations. Formally speaking, this means a decoupling of excitations and de‐excitations, where the excitation solutions are obtained via the Hermitian eigenvalue equation

(10) AX=ωX.

The TDA energy, thus, enters as the leading term into the full TDDFT energy according to Equation (8) assuming that the **Y** amplitudes are small. Practically speaking, the TDA is effective in terms of reducing computational cost as well as having the advantage of being more numerically stable due to it being in the form of a Hermitian eigenvalue problem [3]. In particular, triplet instabilities can be effectively avoided using the TDA [6]. In this context, it is interesting to note that TDA computations can produce negative excitation energies, not running into numerical problems when the closed‐shell and response states cross. The situation is more complicated for full TDDFT where imaginary excitation energies are often encountered near crossings of $S_{0}$ with $T_{1}$ or $S_{1}$.

As opposed to the potential practical advantages of the TDA, one finds that full LR‐TDDFT has clear advantages from a formal perspective in terms of gauge invariance and the fulfillment of sum rules [2, 3, 10]. For example, the Thomas–Reiche–Kuhn sum rule, stating that the sum over all oscillator strengths between the ground and excited states $\left(f_{0I}\right)$ equals the number of electrons $\left(N_{\mathrm{el}}\right)$

(11) $$\sum_{I}f_{0I}=N_{\mathrm{el}}$$

holds for full LR‐TDDFT but not for the TDA. Here, the oscillator strength is defined as

(12) $$f_{0I}=\frac{2}{3}\left(E_{I}-E_{0}\right){\vec{\mu }}_{0I}^{2}$$

where $E_{I}$ and $E_{0}$ are the energies of the two states and ${\vec{\mu }}_{0I}^{2}$ is the transition dipole moment between them. The transition dipole moments, in turn, are evaluated as

(13) $${\vec{\mu }}_{0I}=\sum_{\mathrm{ia}}\left(X_{\mathrm{ia}}+Y_{\mathrm{ia}}\right){\vec{\mu }}_{\mathrm{ia}}$$

where ${\vec{\mu }}_{\mathrm{ia}}$ is the matrix element of the dipole operator evaluated between orbitals i and a. From Equation (13), it can be seen that within full TDDFT there are interference effects between the excitation and de‐excitation contributions. If $X_{\mathrm{ia}}$ and $Y_{\mathrm{ia}}$ have the same sign, then constructive interference is observed, otherwise destructive interference. This can lead to substantial differences between TDDFT and TDA transition moments and will be illustrated below.

Below, we will use the terms “TDDFT” or “full TDDFT” to refer to solutions of Equation (1) whereas “TDA” refers to Equation (10). All discussions will implicitly assume the Kohn‐Sham, linear‐response, and adiabatic approximations. It is the purpose of this work to investigate whether there is physics contained within full TDDFT, and especially the de‐excitations, that cannot be captured within the TDA. For this purpose, we need to investigate if and how de‐excitations arise in wave function‐based computations, and we will, next, turn to the question of how this can be addressed on a rigorous footing.

### 2.2 Defining De‐Excitations Within a Wave Function Based Picture

To provide a rigorous connection between TDDFT and wave function‐based methods, we turn to the 1‐electron transition density matrix (1TDM). We will rely on previously established 1TDM analysis methods [31, 32] presenting the main equations below. The 1TDM between the ground‐state wave function $\Psi_{0}$ and excited‐state wave function $\Psi_{I}$ is defined as

(14) $$D_{\mathrm{pq}}^{0I}=\left\langle \Psi_{0}|a_{p}^{†}a_{q}|\Psi_{I}\right\rangle$$

where $a_{p}^{†}$ and $a_{q}$ are the creation and annihilation operators referring to orbitals $\phi_{p}$ and $\phi_{q}$, respectively. This equation is completely independent of the wave function model and applies for standard single‐reference methods, for multireference methods, and for spin–flip and related methods. 1TDMs can also be computed for methods that do not produce explicit wave functions such as TDDFT [10] (see below) and linear‐response coupled cluster [33], thus providing a very general basis for comparing between different computational methods. In real space, the 1TDM is written as

(15) $$\gamma_{0I}\left(r_{h},r_{e}\right)=\sum_{\mathrm{pq}}D_{\mathrm{pq}}^{0I}\phi_{p}\left(r_{h}\right)\phi_{q}\left(r_{e}\right)$$

where $r_{h}$ and $r_{e}$ denote the coordinates of the excitation hole and excited electron, respectively. The 1TDM, thus, effectively encodes the distribution of the electron–hole pair, providing a solid basis for quantitative and visual analysis methods [34, 35]. Using this construction, we can define operator expectation values of the effective exciton wave function using [35]

(16) $${\left\langle \hat{O}\right\rangle }_{\mathrm{ex}}=\Omega^{-1}\left\langle \gamma_{0I}|\hat{O}|\gamma_{0I}\right\rangle .$$

Note that these expectation values are introduced for computing method‐independent wave function descriptors, but that they do not generally represent physically observable quantities. We start with the normalization factor Ω, defined as

(17) $$\Omega =\left\langle \gamma_{0I}|\gamma_{0I}\right\rangle =\sum_{\mathrm{pq}}{\left|D_{\mathrm{pq}}^{0I}\right|}^{2}=\left(D^{0I}D^{0I,T}\right).$$

For wave function‐based methods, this norm is an interesting descriptor in its own right [36], and can be used to define the doubly excited character of the excitation [31, 37]. For TDDFT, this assignment is not so clear as will be discussed below.

Next, to define de‐excitations in a rigorous way, we will use the particle‐hole permutation operator $P_{\mathrm{he}}$, which by acting on the 1TDM exchanges the coordinates of the hole and electron, that is,

(18) $$P_{\mathrm{he}}\gamma_{0I}\left(r_{h},r_{e}\right)=\gamma_{0I}\left(r_{e},r_{h}\right).$$

Inserting $P_{\mathrm{he}}$ into Equation (16) yields the de‐excitation measure $P_{\mathrm{he}}$ defined as [32]

(19) $$P_{\mathrm{he}}={\left\langle P_{\mathrm{he}}\right\rangle }_{\mathrm{ex}}=\Omega^{-1}\sum_{\mathrm{pq}}D_{\mathrm{pq}}^{0I}D_{\mathrm{qp}}^{0I}=\Omega^{-1}\left(D^{0I}D^{0I}\right)$$

This is analogous to Equation (17), only that the electron and hole indices are swapped for the second matrix. A non‐vanishing value of $P_{\mathrm{he}}$ is obtained if, for example, there is one contribution where the electron is promoted from the HOMO to the LUMO and another configuration where it is “demoted” from the LUMO to the HOMO. We will highlight, below, that $P_{\mathrm{he}}$ is generally consistent with de‐excitations seen in TDDFT.

An interesting property of $P_{\mathrm{he}}$ is that it must vanish for nilpotent matrices. In other words, it always holds that

(20) $$D^{0I}D^{0I}=0\Rightarrow P_{\mathrm{he}}=0$$

It is known that the 1TDM in the case of CIS is nilpotent [38]. The $P_{\mathrm{he}}$ value, thus, provides a measure of deviations from CIS in terms of non‐nilpotency. Note that this is analogous to the widely used practice of considering the non‐idempotency of the ground‐state density matrix to measure deviations from Hartree–Fock theory and, thus, unpaired electrons [11, 12, 13, 14, 15, 16].

$P_{\mathrm{he}}$ values of 0/+1/−1 mean that the 1TDM is nilpotent/symmetric/antisymmetric. It follows that in the case of $P_{\mathrm{he}}=-1/+1$ any matrix element of a symmetric/antisymmetric operator between $\Psi_{0}$ and $\Psi_{I}$ must vanish, providing new effective selection rules to diradical excited states [39]. More generally, positive $P_{\mathrm{he}}$ will result in constructive interference regarding symmetric operators and negative $P_{\mathrm{he}}$ values in destructive interference, and this will be discussed in more detail below in the context of optical transition strengths.

Whereas $P_{\mathrm{he}}$ allows us to quantify de‐excitations as such, we will also want to obtain some more insight about what they tell us about the underlying wave functions. To do so, we will use the electron–hole correlation coefficient ($R_{\mathrm{eh}}$) [35]. Using the exciton expectation value expression of Equation (16), this can be defined in analogy to Pearson's correlation coefficient as

(21) $$R_{\mathrm{eh}}=\frac{{\left\langle r_{h}r_{e}\right\rangle }_{\mathrm{ex}}-{\left\langle r_{h}\right\rangle }_{\mathrm{ex}}{\left\langle r_{e}\right\rangle }_{\mathrm{ex}}}{\sigma_{h}\sigma_{e}}$$

where the root‐mean‐square hole size is defined as

(22) $$\sigma_{h}=\sqrt{{\left\langle {r_{h}}^{2}\right\rangle }_{\mathrm{ex}}-{\left\langle r_{h}\right\rangle }_{\mathrm{ex}}^{2}}$$

and analogously for the electron size $\sigma_{e}$.

The value of $R_{\mathrm{eh}}$ is zero for simple orbital‐to‐orbital transitions. In the case of a standard closed‐shell molecule, one finds that a positive value of $R_{\mathrm{eh}}$ means enhanced local character, as seen for example in the case of bound excitons [40]. Conversely, a negative value of $R_{\mathrm{eh}}$ can be associated to dynamic charge transfer character [35]. Similar relations also hold for an open‐shell ground state only that $R_{\mathrm{eh}}$ now reflects correlations in both ground and excited states [39], as discussed in some more detail below.

Finally, we will also use the exciton size [34]

(23) $$d_{\mathrm{exc}}=\sqrt{{\left\langle {\left|r_{e}-r_{h}\right|}^{2}\right\rangle }_{\mathrm{ex}}}$$

measuring the root‐mean‐square electron–hole separation. The $d_{\mathrm{exc}}$ value can be used as a charge transfer diagnostic for TDDFT [40, 41], applicable even in highly symmetric systems such as pQDM. Values of $d_{\mathrm{exc}}$ below 4 Å are generally associated with locally excited states, whereas higher values indicate at least partial charge transfer. Note that due to the small size of pQDM, we use $d_{\mathrm{exc}}$ rather than the fragment‐based analysis in TheoDORE [42], which otherwise serves a similar purpose.

### 2.3 Analysis of TDDFT Computations

Following the general definitions of our wave function analysis approaches, we can now proceed to the specific case of TDDFT. Indeed, to use the above formalism, one only has to define the 1TDM, which in TDDFT can be written as [3, 10, 41]

(24) $$\gamma_{0I}\left(r_{h},r_{e}\right)=\sum_{\mathrm{ia}}X_{\mathrm{ia}}\phi_{i}\left(r_{h}\right)\phi_{a}\left(r_{e}\right)+\sum_{\mathrm{ia}}Y_{\mathrm{ia}}\phi_{a}\left(r_{h}\right)\phi_{i}\left(r_{e}\right).$$

Note that in the first part of the equation, the hole resides in the occupied orbitals and the electron in the virtual orbitals, whereas the relation is reversed in the second part, marking the de‐excitations. Expressed in matrix form, one obtains, in line with Equation (5), the expression

(25) $$D_{\mathrm{ia}}^{0I}=X_{\mathrm{ia}}$$

for the occupied–virtual block of the 1TDM and

(26) $$D_{\mathrm{ai}}^{0I}=Y_{\mathrm{ia}}$$

for the virtual–occupied block. Inserted into Equation (17), this yields for the normalization factor Ω

(27) $$\Omega =||X|{|}^{2}+||Y|{|}^{2}=1+2||Y|{|}^{2}.$$

Note that this implies that Ω is always greater than or equal to one in the case of full TDDFT. This is opposed to wave function‐based methods where Ω is essentially always lower than one [37], and serves as a measure for doubly excited character. The difference derives from the fact that within TDDFT, the normalization condition of Equation (9) is used rather than requiring a normalized wave function within Equation (5). All other descriptors to follow are normalized by Ω, meaning that the overall normalization does not affect the results. However, we note here that Ω as such cannot be compared between wave function‐based methods and TDDFT.

Moving on, we can now evaluate the de‐excitation measure $P_{\mathrm{he}}$ via Equation (19) to obtain

(28) $$P_{\mathrm{he}}=\frac{\sum_{\mathrm{ia}}X_{\mathrm{ia}}Y_{\mathrm{ia}}}{||X|{|}^{2}+||Y|{|}^{2}}$$

The $P_{\mathrm{he}}$ value is, thus, a measure of the X/Y cross terms (see also Reference [9]). It strictly vanishes in the case of configuration interaction singles or the TDA. In the case of TDDFT, $P_{\mathrm{he}}$ can deviate from zero only if there are contributions from $Y_{\mathrm{ia}}$ amplitudes. More specifically, a non‐vanishing $P_{\mathrm{he}}$ value is obtained whenever there are excitations and de‐excitations involving the same pair of orbitals.

Equation (28) can be simplified in the case of only small de‐excitation contributions. If we make the approximation that $Y_{\mathrm{ia}}\approx \epsilon X_{\mathrm{ia}}$ for some small value of ϵ with $\epsilon^{2}\approx 0$, then we find that

(29) $$P_{\mathrm{he}}\approx ||Y||$$

Thus, roughly speaking, $P_{\mathrm{he}}$ does indeed reflect the norm of the de‐excitation vector (||Y||). Note, however, that using $P_{\mathrm{he}}$ has two clear advantages over simply using ||Y||: (i) it is well‐defined for any computational method producing 1TDMs and (ii) it also includes information about relative signs. Both will be exemplified in more detail below.

### 2.4 Model Wave Functions

Before showing concrete results, from realistic computations, we want to briefly comment on the expected results based on a simple two‐orbital two‐electron model (TOTEM) applied to the case of a molecule with partial diradical character [39] (following also References [43, 44, 45]). Within the TOTEM, the three relevant states for the following discussion are the singlet ground state $\Psi_{0}$, the diradical triplet state $\Psi_{T}$, and the zwitterionic singlet state $\Psi_{Z}$, defined as

(30) $$\left|\Psi_{0}\right\rangle =\cos\left(\eta \right)\left|\phi_{H}{\bar{\phi }}_{H}\right\rangle -\sin\left(\eta \right)\left|\phi_{L}{\bar{\phi }}_{L}\right\rangle$$

(31) $$\left|\Psi_{T}\right\rangle =\frac{1}{\sqrt{2}}\left(\left|\phi_{H}{\bar{\phi }}_{L}\right\rangle -\left|\phi_{L}{\bar{\phi }}_{H}\right\rangle \right)$$

(32) $$\left|\Psi_{Z}\right\rangle =\frac{1}{\sqrt{2}}\left(\left|\phi_{H}{\bar{\phi }}_{L}\right\rangle +\left|\phi_{L}{\bar{\phi }}_{H}\right\rangle \right)$$

where $\phi_{H}$ and $\phi_{L}$ are the frontier MOs involved and the bar indicates spin‐down. The parameter η is the degree of mixing between the configurations and runs from 0 to π/4; η=0 corresponds to a closed‐shell molecule with doubly occupied HOMO, whereas η=π/4 marks the perfect diradical case. Notably, in this model, the ground‐state wave function $\Psi_{0}$ varies with η, whereas $\Psi_{T}$ and $\Psi_{Z}$ are always simple HOMO/LUMO states. The states $\Psi_{T}$ and $\Psi_{Z}$ are assigned as having “diradical” and “zwitterionic” characters, respectively. This assignment follows from a valence‐bond analysis of these wave functions [46, 47, 48, 49] and applies to HOMO/LUMO valence excited states of many *π*‐conjugated molecules.

Within the developed model, considering the de‐excitation measure $P_{\mathrm{he}}$ evaluated for the transition from the ground state to the states $\Psi_{T}$ and $\Psi_{Z}$, one obtains the following relation between $P_{\mathrm{he}}$ and the mixing angle η [39]

(33) $$\Psi_{0}\to \Psi_{T}:P_{\mathrm{he}}=+\sin\left(2\eta \right)$$

(34) $$\Psi_{0}\to \Psi_{Z}:P_{\mathrm{he}}=-\sin\left(2\eta \right)$$

First, we note that these expressions do concern not only the magnitude of $P_{\mathrm{he}}$ but also its sign; one expects positive $P_{\mathrm{he}}$ values for diradical triplets and negative values for zwitterionic singlets. Below, we will compare these model predictions with the results from realistic computations.

The $P_{\mathrm{he}}$ value is zero if η is zero (i.e., in the closed‐shell case) and its absolute value goes up of η increases. Note that, within the developed model, the excited‐state wave functions are independent of η. This means the $P_{\mathrm{he}}$ value is actually a reflection of the change in the ground‐state wave function where an increase in $P_{\mathrm{he}}$ goes along with increased open‐shell character of the ground state. To investigate this connection in some more detail, we will also investigate the $y_{0}$ descriptor [50, 51], defined as the occupation number of the first weakly occupied natural orbital. Within the model, it is given as

(35) $$y_{0}=2\sin^{2}\eta$$

Comparing Equations (35) and (33) shows that, within the model, there is a direct connection between $P_{\mathrm{he}}$ and $y_{0}$, given by the relation

(36) $$y_{0}=1-\sqrt{1-{P_{\mathrm{he}}}^{2}},$$

which will be investigated for realistic computations below. In the case of small $P_{\mathrm{he}}$ values, the relation can be approximated as

(37) $$y_{0}\approx \frac{{P_{\mathrm{he}}}^{2}}{2}$$

highlighting that $P_{\mathrm{he}}$ increases monotonically with increasing $y_{0}$. Here, $P_{\mathrm{he}}$ is particularly sensitive to smaller numbers of unpaired electrons, considering that $y_{0}$ is proportional to its square. Nonetheless, at least within the model, there is a one‐to‐one correspondence between $y_{0}$ and $P_{\mathrm{he}}$ and one may use either one to define diradical character.

### 2.5 Computational Details

Excited states based on DFT were computed using full TDDFT as well as the TDA [1, 5] using the PBE and PBE0 functionals along with the def2‐TZVP basis set [52, 53, 54]. All TDA and TDDFT computations were based on a spin‐restricted Kohn–Sham (RKS) reference. For comparison, DFT triplet energies were also computed using unrestricted Kohn–Sham (UKS) theory (not using TDA or TDDFT). These computations were carried out using Q‐Chem 6.1 [55].

CASSCF/CASPT2 results were taken from Ref. [39]. In brief, the CASSCF calculations used an active space of 8 electrons in 8 active orbitals applying the ANO‐S‐VDZP basis set [56, 57]. Dynamic correlation was treated with multi‐state second‐order perturbation theory (MS‐CASPT2) [58], with an IPEA shift [59] of 0.25 and a regularization parameter [60] $\sigma^{2}$ of 0.3.

Wave function analysis to compute Ω, $P_{\mathrm{he}}$, $R_{\mathrm{eh}}$, $d_{\mathrm{exc}}$ as well as NTOs was performed using the wave function analysis library libwfa [61] employing its OpenMolcas and Q‐Chem interfaces [31, 62]. In the case of TDDFT, the 1TDMs are derived from the **X** and **Y** vectors following Equation (24). In the case of MS‐CASPT, the 1TDMs are computed following Equation (14) using as input CASSCF wave functions mixed via the multi‐state procedure but not including explicit PT2 corrections. Some post‐processing was done using TheoDORE 3.1.1 [42]. As a technical note, it should be pointed out that TheoDORE by itself is currently not capable to compute $P_{\mathrm{he}}$ values from TDDFT and users are advised to use the Q‐Chem/libwfa implementation where it is assured that the **X** and **Y** vectors are interpreted consistently with Equation (24).

## 3 Results and Discussion

### 3.1 pQDM—Planar Geometry

We start the discussion with pQDM at its planar geometry optimized in its lowest triplet state [39]. Basic results for TDDFT/PBE0 and TDA/PBE0 computations are presented in Table 1. Starting with TDDFT/PBE0, we find that the lowest state is a triplet of $A_{u}$ symmetry ($1^{3}A_{u}$) lying at around 1.2 eV. In line with Reference [49] it can be assigned as the 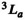 state. This state is dominated by the HOMO/LUMO excitation with an excitation amplitude of $x_{\mathrm{HL}}=1.07$. Interestingly, there is also a pronounced HOMO/LUMO de‐excitation contribution ($y_{\mathrm{HL}}=0.45$). In addition, there are secondary contributions involving the HOMO‐1 and LUMO+1 where, again, excitations and de‐excitations contribute ($x_{H_{1}L_{1}}$/$y_{H_{1}L_{1}}$ = 0.19/0.12). The next state (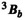) involves HOMO‐1/LUMO and HOMO/LUMO+1 excitations in about equal mixture. These are dominated by excitations, accompanied by non‐vanishing de‐excitations. The first singlet (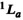), lying at 4.26 eV, is the counterpart to the 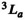 state. It is the only bright state considered here. Its response vector is dominated by the HOMO/LUMO excitation $\left(x_{\mathrm{HL}}=0.99\right)$ with additional contributions from the HOMO/LUMO de‐excitation $\left(y_{\mathrm{HL}}=-0.18\right).$ Comparing the $y_{\mathrm{HL}}$ de‐excitation contributions of the 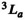 and 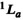 states, we make two observations: (i) they are significantly more pronounced for the triplet and (ii) $x_{\mathrm{HL}}$ and $y_{\mathrm{HL}}$ are both positive for the triplet whereas they have opposite signs for the singlet. The more pronounced values for the triplet can be related to an emerging triplet instability. The sign change between $x_{\mathrm{HL}}$ and $y_{\mathrm{HL}}$ is expected to lead to destructive interference following Equation (13), thus, lowering transition moments. Both phenomena will be revisited in more detail below. The final state shown 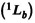 is a mixture of the HOMO‐1/LUMO and HOMO/LUMO+ 1 excitations similar to the 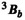 state, but with opposite relative sign between the two configurations. Interestingly, we notice that the de‐excitation contributions vanish almost completely in the case of this state.

**TABLE 1:** Vertical excitation energies $\left(\Delta E,\mathrm{eV}\right),$ oscillator strengths (f), and excitation/de‐excitation amplitudes ($x_{\mathrm{ia}}$/$y_{\mathrm{ia}}$) computed for planar pQDM at its triplet‐optimized geometry using TDDFT/PBE0 and TDA/PBE0.

Method	State	Δ*E*	*f*	$x_{HL}$/$y_{HL}$	$x_{H_{1}L}$/$y_{H_{1}L}$	$x_{{\mathrm{HL}}_{1}}$/$y_{{\mathrm{HL}}_{1}}$	$x_{H_{1}L_{1}}$/$y_{H_{1}L_{1}}$
TDDFT	1^3^ *A*_*u*_/^3^ *L*_*a*_	1.202	—	1.07/0.45			0.19/0.12
	1^3^ *B*_*g*_/^3^ *B*_*b*_	3.368	—		0.72/0.10	0.67/0.09	
	1^1^ *A*_*u*_/^1^ *L*_*a*_	4.264	0.756	0.99/−0.18			–0.18/–0.05
	1^1^ *B*_*g*_/^1^ *L*_*b*_	4.652	—		−0.50/0.00	0.87/0.00	
TDA	1^3^ *A*_*u*_/^3^ *L*_*a*_	1.869	—	0.98			0.12
	1^3^ *B*_*g*_/^3^ *B*_*b*_	3.630	—		0.72	0.66	
	1^1^ *A*_*u*_/^1^ *L*_*a*_	4.785	1.164	0.93			−0.31
	1^1^ *B*_*g*_/^1^ *L*_*b*_	4.659	—		−0.55	0.83	

Moving to the TDA/PBE0 results, we find that all excited states are raised in energy with the biggest gain (>0.6 eV) for the 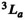 state, which was the state with the largest de‐excitation contributions. Conversely, the 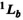 state stays almost unaltered considering that also its de‐excitations vanish. Notably, there is a swap in state ordering as the 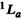 state moves up in energy but, otherwise, the qualitative discussion remains unaltered.

Viewing Table 1, two questions come to mind comparing TDDFT and TDA. First, which method produces more accurate energies? Second, which method produces more accurate 1TDMs capturing the underlying physics more accurately? To answer these questions, we include high‐level multireference results as initially presented in Reference [39]. Before proceeding, we note that neither of the methods used is without pitfalls [63, 64, 65]. Aside from the diradical character, which is the main area to be investigated within this work, TDDFT and TDA have well‐known problems with charge transfer and bound exciton states [40, 66, 67]. Conversely, CASSCF has notorious problems in describing zwitterionic states [68, 69], such as the 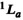 state considered here. However, the problems of CASSCF and TDDFT/TDA are largely complementary meaning that it is reasonable to trust the results if they are consistent. Moreover, here we present MS‐CASPT2 energies, which are generally considered more reliable provided that an appropriate CASSCF starting point is chosen. As shown in Table 2, the first two triplet states from CASPT2 lie at 2.3 and 4.1 eV, notably higher than both TDDFT and TDA, with the TDA being somewhat closer to the CASPT2 reference. The singlet states lie at 4.7 and 4.8 eV, very close to the TDA results and somewhat above the TDDFT results. In terms of vertical excitation energies we, thus, find that the TDA outperforms TDDFT.

**TABLE 2:** Analysis of planar pQDM at the CASPT2, TDDFT/PBE0, and TDA/PBE0 levels: Vertical excitation energies (ΔE, eV), oscillator strengths (f), 1TDM norms (Ω), de‐excitation measures ($P_{\mathrm{he}}$), correlation coefficients ($R_{\mathrm{eh}}$), and exciton sizes ($d_{\mathrm{exc}}$, Å).

Method	State	Δ*E*	*f*	Ω	*P*_*he*_	*R*_*eh*_	*d*_*exc*_
CASPT2	1^3^ *A*_*u*_/^3^ *L*_*a*_	2.279	—	0.930	0.381	0.353	2.969
	1^3^ *B*_*g*_/^3^ *B*_*b*_	4.137	—	0.839	0.263	0.264	2.552
	1^1^ *A*_*u*_/^1^ *L*_*a*_	4.784	1.447	0.843	−0.265	−0.048	3.806
	1^1^ *B*_*g*_/^1^ *L*_*b*_	4.677	—	0.773	−0.019	−0.059	3.337
TDDFT	1^3^ *A*_*u*_/^3^ *L*_*a*_	1.202	—	1.494	0.731	0.471	2.627
	1^3^ *B*_*g*_/^3^ *B*_*b*_	3.368	—	1.063	0.305	0.246	2.621
	1^1^ *A*_*u*_/^1^ *L*_*a*_	4.264	0.756	1.083	−0.288	−0.140	3.918
	1^1^ *B*_*g*_/^1^ *L*_*b*_	4.652	—	1.001	−0.026	−0.061	3.328
TDA	1^3^ *A*_*u*_/^3^ *L*_*a*_	1.869	—	1.000	0.000	0.108	3.454
	1^3^ *B*_*g*_/^3^ *B*_*b*_	3.630	—	1.000	0.000	0.157	2.811
	1^1^ *A*_*u*_/^1^ *L*_*a*_	4.785	1.164	1.000	0.000	−0.024	3.661
	1^13^ *B*_*g*_/^1^ *L*_*b*_	4.659	—	1.000	0.000	−0.072	3.340

Having discussed the energies, we now proceed to the wave function descriptors from Section 2.2, with results presented in Table 2. We start with the norm of the 1TDM (Ω). For wave function‐based methods, Ω is essentially always less than or equal to 1 where values significantly lower than 1 imply doubly excited character [37]. For the CASPT2 states, we always find that Ω>0.75 indicating singly excited character for all states considered. The interpretation of Ω in the case of TDDFT is different as it only reflects the normalization condition of Equation (9) via Equation (27). For TDDFT, we find that the 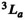 state, possessing significant de‐excitation character, has an Ω value much larger than 1, whereas values only slightly above 1 are obtained for the other states. For the TDA, by contrast Ω is always exactly 1 by construction. Out of the 1TDM descriptors discussed above, Ω sticks out as it is difficult to generalize between wave function methods and TDDFT. Conversely, the de‐excitation measure ($P_{\mathrm{he}}$), correlation coefficient ($R_{\mathrm{eh}}$), and exciton size ($d_{\mathrm{exc}}$) are normalized by Ω and can, thus, be directly compared. Viewing the de‐excitations, we find that $P_{\mathrm{he}}$ is markedly positive for the first two triplet states, whereas it becomes negative for the 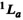 state and is close to zero for the 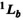 state. This trend reflects Equation (33) and (34) and is nicely reproduced between the CASPT2 and TDDFT methods underscoring the physics contained in the de‐excitations. The $P_{\mathrm{he}}$ values are per construction zero at the TDA level and, hence, no interesting information can be discerned here.

To gain some more insight into the underlying 1TDMs, we compute the correlation coefficient ($R_{\mathrm{eh}}$) giving information about the concerted motion between the electron and hole quasiparticles [35, 40]. We will discuss this quantity in some more detail below. But for now it is enough to realize that, again, we obtain consistent results between the different methods where the triplets experience positive correlation and the singlets negative correlation. This trend is reproduced with TDDFT and TDA, where the TDDFT values are clearly closer to the CASPT2 reference.

Finally, the exciton sizes ($d_{\mathrm{exc}}$), representing charge transfer character, are presented in Table 2. Viewing the CASPT2 results, we find that the $d_{\mathrm{exc}}$ values for the triplet states are always below 3 Å, whereas they are above 3 Å for the singlet states, a difference arising due to the exchange repulsion experienced by the singlets (cf. References [70, 71]). Notably, the positive $R_{\mathrm{eh}}$ values represent dynamic exciton binding and are, thus, associated with lower $d_{\mathrm{exc}}$ values (cf. Reference [35]). Viewing the TDDFT and TDA results, we find that the $d_{\mathrm{exc}}$ values from CASPT2 are generally well reproduced (with the exception of the 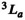 state using TDA/PBE0). Moreover, all $d_{\mathrm{exc}}$ values are below the threshold of 4 Å, thus indicating purely locally excited states. During, the twisting process, as studied in more detail below, the $d_{\mathrm{exc}}$ values slightly increase but always stay below 5.1 Å. These low $d_{\mathrm{exc}}$ values suggest that charge transfer is not a primary concern here.

**FIGURE 2:**
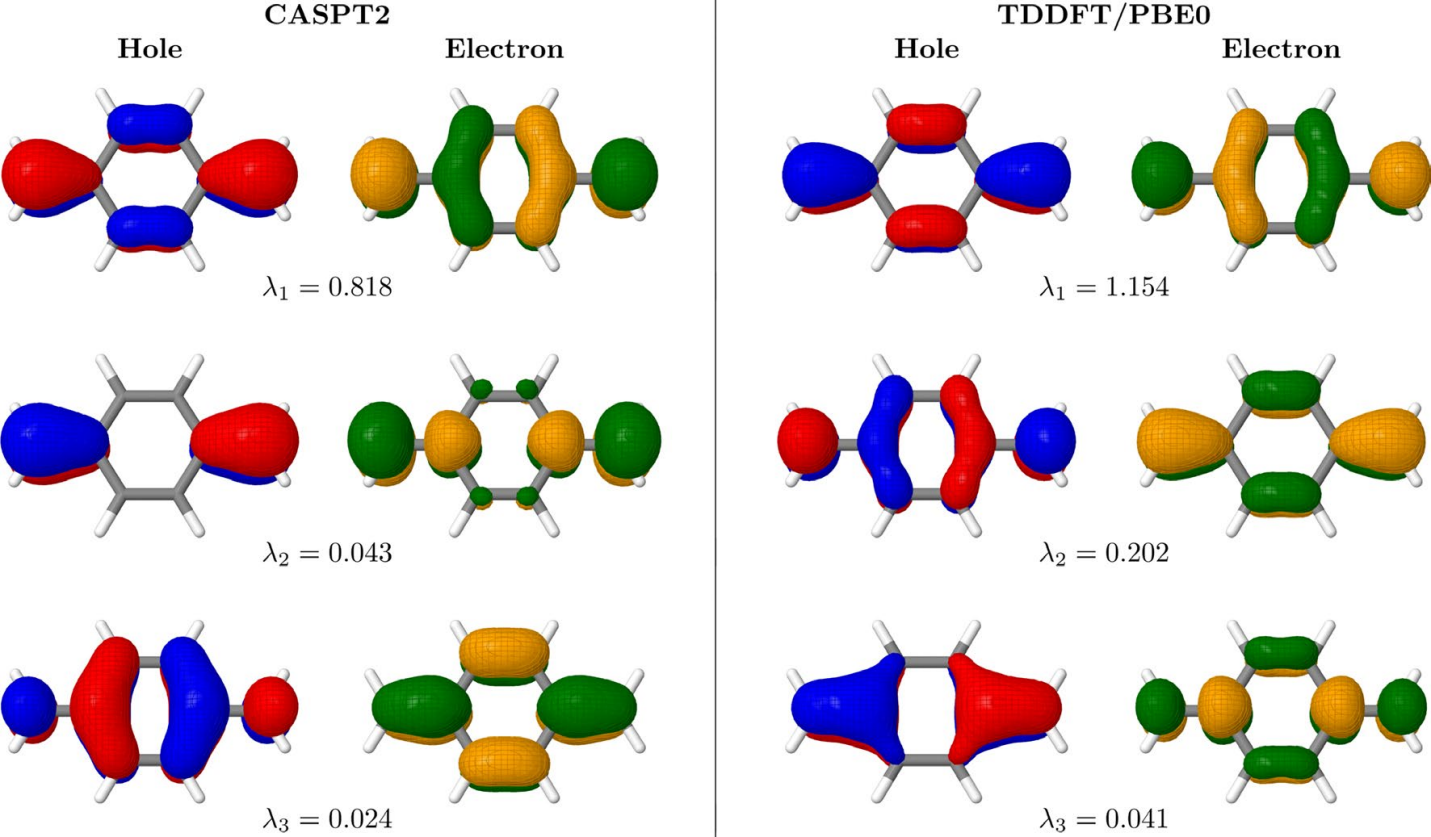
Natural transition orbitals for the $T_{1}$ state of pQDM computed at the CASPT2 (left) and TDDFT/PBE0 (right) levels of theory, considering the planar triplet‐optimized geometry. Amplitudes ($\lambda_{i}$) are given below the respective orbital pair.

Figure 2 presents the $T_{1}$ state natural transition orbitals (NTOs) computed at the CASPT2 and TDDFT/PBE0 levels and we will scan these for signatures of the de‐excitations. We find that, in both cases, the dominant pair of NTOs reflects the HOMO/LUMO transition as expected for this state. At the CASPT2 level, the next pair (with a contribution of $\lambda_{2}=0.043$) derives from a different orbital pair with the symmetry along the long molecular axis reversed. Crucially, the third contribution ($\lambda_{2}=0.024$) is the same as the first one only that electron and hole are swapped. The electron now resides in the HOMO whereas the hole is in the LUMO. This represents the de‐excitation contributions in a pictorial way and shows that de‐excitations arise naturally within CASPT2. The TDDFT/PBE0 NTOs reflect the CASPT2 ones, only that the LUMO/HOMO de‐excitation becomes the second most important contribution ($\lambda_{2}=0.202$) in line with its enhanced de‐excitation character shown in Table 2. Reviewing Table 1, we find that the NTO amplitudes $\lambda_{1}$ and $\lambda_{2}$ are approximately equal to the squares of the $x_{\mathrm{HL}}$ and $y_{\mathrm{HL}}$ values for TDDFT, bridging between the different representations.

To summarize, the above discussion has shown that de‐excitations arise naturally in wave function‐based theories along with TDDFT as long as one uses appropriate 1TDM definitions, that is, Equations (14) and (24), and analyses the results accordingly. De‐excitations were quantified using $P_{\mathrm{he}}$ values and represented pictorially using NTOs, always finding consistent trends. In the discussion to follow, we will now endeavor to obtain more insight into the phenomenon and, particularly, how de‐excitations change once the diradical character increases in the model of twisted pQDM.

### 3.2 pQDM—Potential Curve

**FIGURE 3:**
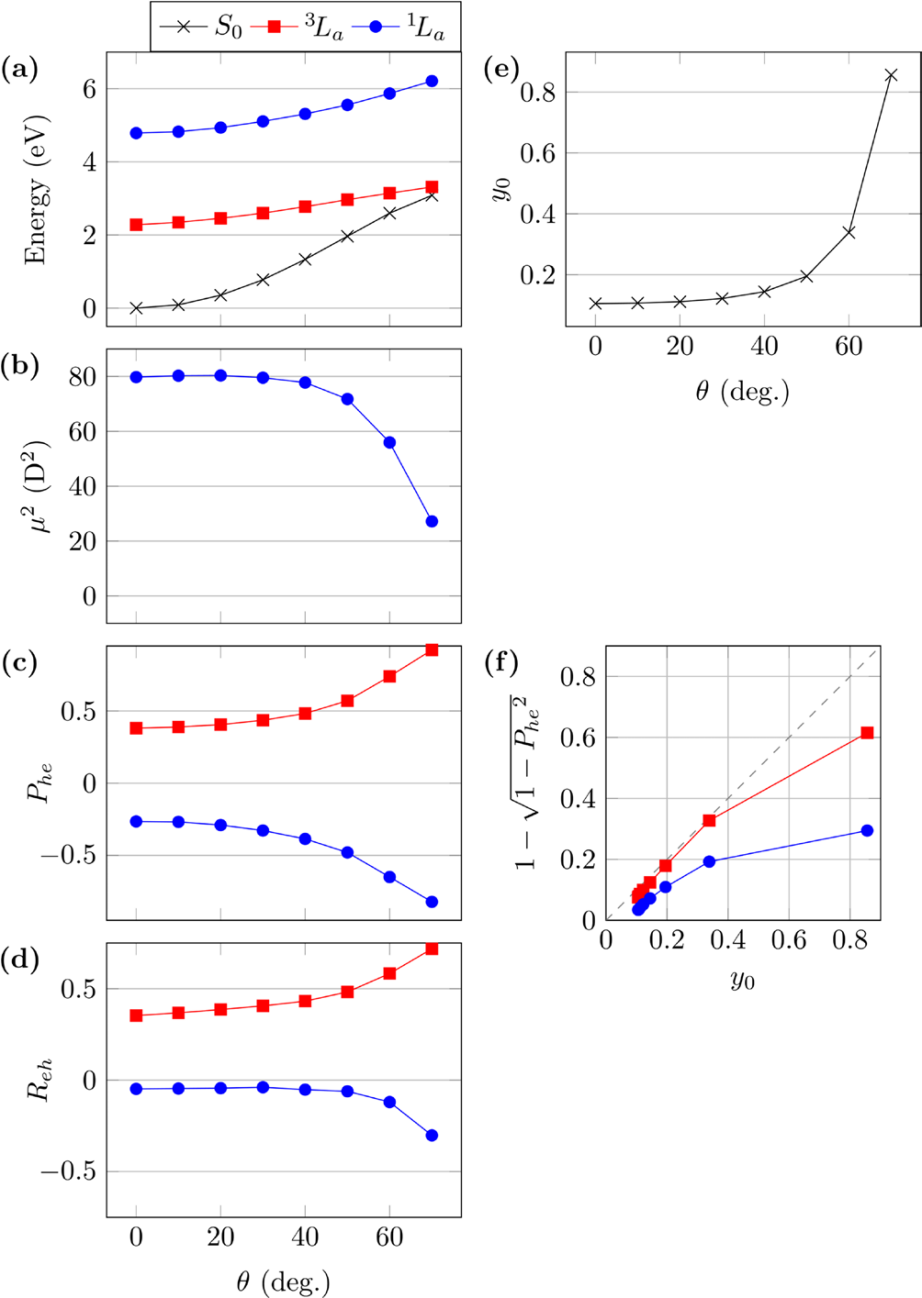
Analysis of twisting in pQDM at the MS‐CASPT2 level considering the $S_{0}$, 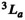, and 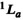 states: (a) energies, (b) squared transition dipole moment to 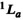, (c) de‐excitations ($P_{\mathrm{he}}$), (d) correlation coefficient ($R_{eh}$), (e) diradical character of $S_{0}$ ($y_{0}$), and (f) correlation between $y_{0}$ and $P_{\mathrm{he}}$.

Having discussed the vertical excitation energies at the equilibrium geometry, it is now interesting to study how the states and their energies change upon twisting. Here, pQDM is a convenient model system where the diradical character can be increased with enhanced twisting [39]. We start with the CASPT2 results; Figure 3 shows computational results for varying torsion angles θ. Results are presented for the $S_{0}$ state, the lowest triplet (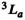), and the first bright singlet (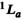). These reflect the $\Psi_{0}$, $\Psi_{T}$, $\Psi_{Z}$ states of the model developed above. A more complete description, including all low‐energy states is presented in Reference [39]. Starting with the energies (Figure 3a), we note that these are all well‐spaced out at the planar geometry (θ=0) with 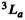 and 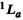 vertical excitation energies of 2.28 and 4.78 eV, respectively. Upon twisting, the energies of all states go up and this increase is particularly pronounced for $S_{0}$ meaning that it becomes near‐degenerate with 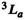 at around $\theta =70^{{}^{\circ}}$. Conversely, the gap between 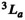 and 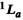 remains fairly constant.

We next turn to the optical brightness. For this purpose, we present the squared transition dipole moment ($\mu^{2}$). Note that $\mu^{2}$ multiplied by the energy gap produces the oscillator strength $\left(f\right)$ as given in Equation (12). We choose $\mu^{2}$ rather than f for our analysis to separate the effects deriving from changes in wave functions from the more trivial effects deriving from changes in the energy gap. As shown in Figure 3b, the $\mu^{2}$ value starts at about 80 D^2^ and experiences a pronounced decline starting at around $\theta =40^{{}^{\circ}}$. The origin of this will be discussed in some more detail below.

Results for the de‐excitation measure $P_{\mathrm{he}}$ are shown in Figure 3c. First we note that, in line with the developed model, Equations (33) and (34), this value is always positive for the triplet and negative for the singlet. The $P_{\mathrm{he}}$ values for singlet and triplet remain fairly constant up until $\theta =40^{{}^{\circ}}$, at which point they start curving out. Within the developed model the absolute $P_{\mathrm{he}}$ values would always be equal for singlet and triplet whereas they are always slightly less for the singlet in practice. This highlights that the TOTEM does not account for all details involved while the overall physics is indeed captured well.

To explain the drastic decline in $\mu^{2}$ values, it is necessary to have a closer look at the connection between these and the $P_{\mathrm{he}}$ values. An enhanced $P_{\mathrm{he}}$ value means, per definition, that excitations come paired with de‐excitations involving the same orbital pair. The transition dipole moment associated to an i→a excitation is equivalent to that of the analogous a→i de‐excitation, considering that these are matrix elements of a symmetric operator. If those are now coupled with opposite signs, as is the case for the 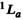 state, then this results in destructive interference for the overall transition dipole moment. As alluded to above, in the limiting case of $P_{\mathrm{he}}=-1$, the 1TDM is an antisymmetric matrix meaning its trace with the symmetric dipole matrix must vanish making the state effectively optically forbidden. Reviewing Figure 3, we note that, indeed, the $\mu^{2}$ values decline just as the $P_{\mathrm{he}}$ values curve out highlighting the connection between the two quantities.

To obtain more insight into the different wave functions, we also compute the electron–hole correlation coefficient as defined in Equation (21). The results are presented in Figure 3d. Importantly, we consistently find a positive correlation for the 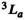 state and a negative correlation for the 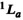 state. As discussed in more detail in Reference [39], the positive $R_{\mathrm{eh}}$ value between $S_{0}$ and 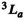 means that both are of a similar character (both are diradicals). Conversely, the negative value for 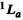 reflects its zwitterionic character. In line with the other curves, one finds that $R_{\mathrm{eh}}$ is fairly steady at first and curves out after a θ value of around $45^{{}^{\circ}}$.

The diradical character of the ground state $y_{0}$ is presented in Figure 3e. It reflects the trends of the other descriptors in the sense that it stays fairly constant at first and shoots up starting around $\theta =40^{{}^{\circ}}$. With $P_{\mathrm{he}}$ and $y_{0}$ computed, it is now of interest whether they are indeed connected via Equation (36). For this purpose, we plot the right‐hand side of Equation (36) against $y_{0}$ in Figure 3f. This plot highlights almost perfect agreement for the triplet, except for the last point. For the singlet, the predicted $y_{0}$ are always lower than actual ones but also monotonically increasing. The results of Figure 3f suggest that it is indeed possible to use $P_{\mathrm{he}}$ computed for the 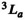 or 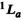 state as a substitute for $y_{0}$ to quantify diradical character.

**FIGURE 4:**
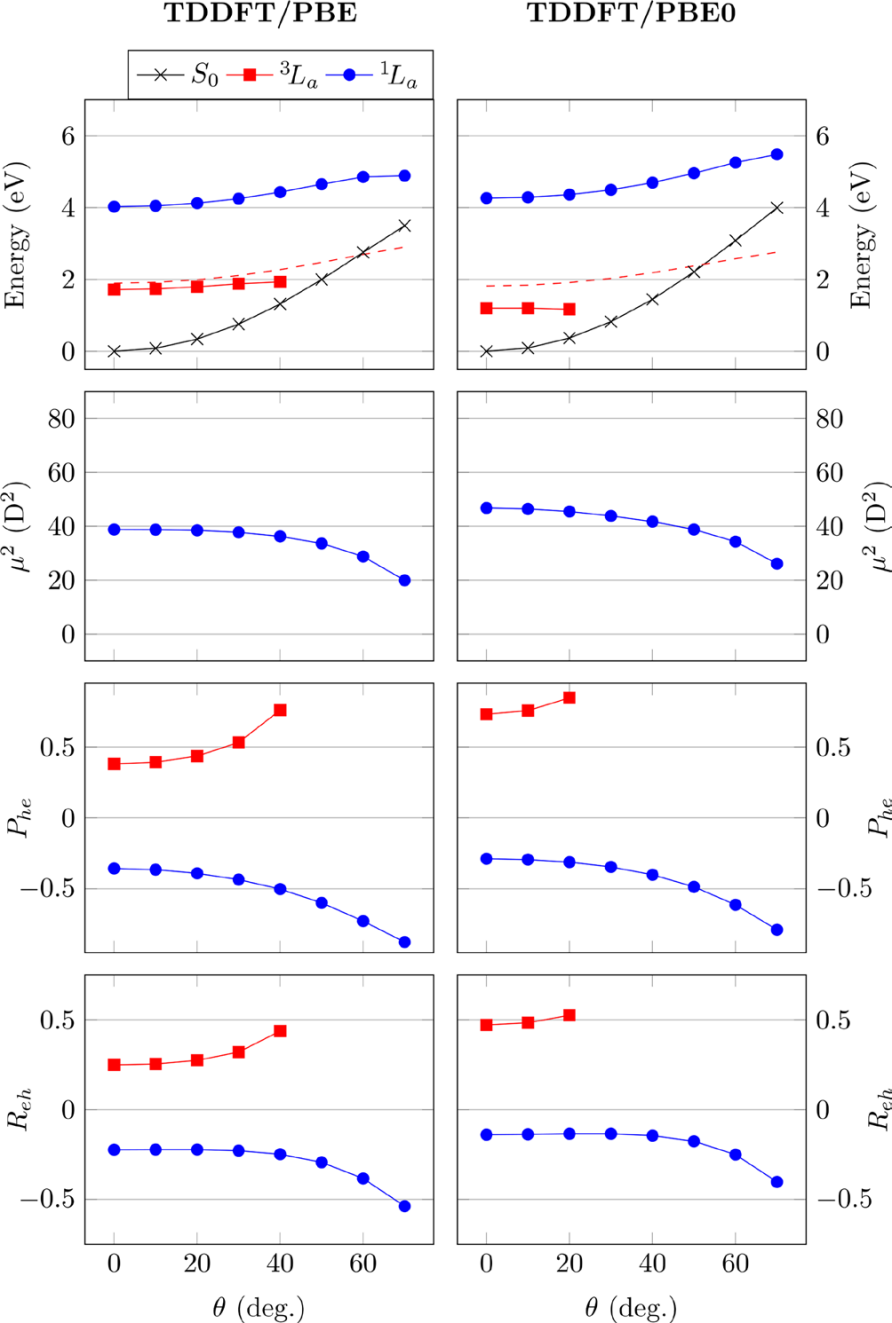
Analysis of twisting in pQDM at the TDDFT/PBE and TDDFT/PBE0 levels: Energies (with triplet UKS energies shown as dotted lines), squared transition dipole moments $\left(\mu^{2}\right),$ de‐excitation measures ($P_{\mathrm{he}}$), and electron–hole correlation coefficients ($R_{\mathrm{eh}}$).

Having presented the CASPT2 results, it is of interest whether the same physics can be qualitatively reproduced by TDDFT. We will investigate energies, transition dipole moments as well as $P_{\mathrm{he}}$ and $R_{\mathrm{he}}$ values, and the results are shown in Figure 4. Starting with full TDDFT in connection with the PBE functional (upper left), one finds that the vertical excitation energies (1.72/4.03 eV for 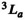/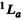) are reasonably well reproduced compared to CASPT2, only that they are somewhat too low. When twisting the molecule, the $S_{0}$ state goes up somewhat more strongly compared to CASPT2 whereas the excited states are more flat. As a consequence, the 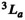 state crosses too early with $S_{0}$ (around $\theta =50^{{}^{\circ}}$) at which point the TDDFT equations become numerically unstable for the triplet. Furthermore, we find that the 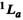 state approaches the $S_{0}$ state more closely after twisting. Using the hybrid PBE0 functional (Figure 4, right) we find a similar shape of the potential surfaces as for PBE only that the 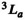 state is even lower. As a consequence, the computation becomes numerically unstable and we obtain results only until $\theta =20^{{}^{\circ}}$. The fact that hybrid functionals exacerbate triplet instabilities has been noted before [6] and can be understood by viewing Equation (3), which highlights the additional exchange‐like coupling term proportional to $c_{\mathrm{HF}}$. Finally, the UKS triplet energies are presented in the top panels of Figure 4 as dashed lines. We note that these are stable over the whole range considered producing values in good agreement with CASPT2.

Moving to the transition dipole moments $\left(\mu^{2}\right),$ we find that TDDFT/PBE and TDDFT/PBE0 both reproduce the appropriate decline in $\mu^{2},$ which, as discussed above, is a signature of the de‐excitations. The overall effect for TDDFT is less pronounced than in the case of CASPT2 but, crucially, the correct qualitative physics is captured. As a more detailed comment it may be noted that the 1TDMs from CASSCF, which are used as input for the CASPT2 transition moments, do not capture crucial effects from *σ*‐electron correlation [32, 69]. Therefore, it is certainly reasonable to assume that the $\mu^{2}$ values from CASSCF/CASPT2 are somewhat overestimated underscoring the validity of the TDDFT values.

Proceeding to the de‐excitation measures $P_{\mathrm{he}}$, we find a similar trend as for CASPT2, considering both TDDFT/PBE and TDDFT/PBE0. Importantly, $P_{\mathrm{he}}$ is always positive for 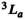 and always negative for 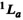, and the lines curve out with increasing torsion. The absolute values of $P_{\mathrm{he}}$ are always somewhat larger for TDDFT than for CASPT2 and this is particularly true for the 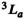 state at the PBE0 level, reflecting its very low energy and borderline triplet instability. Nonetheless, the good qualitative agreement of the $P_{\mathrm{he}}$ values with the model and the CASPT2 results allows one to speculate that, indeed, TDDFT “knows” about the magnitude and other details of the underlying ground‐state correlation. Indeed, the $P_{\mathrm{he}}$ values of the 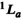 state using TDDFT with the two functionals closely reflect the results from CASPT2. The $P_{\mathrm{he}}$ values from CASPT2, in turn, reflect the ground‐state diradical character $y_{0}$. In summary, this means that $P_{\mathrm{he}}$ values from standard TDDFT computations can be used to approximate the diradical character as computed within sophisticated multireference methods. Thus, $P_{\mathrm{he}}$ can be seen as a diagnostic for diradical character in its own right. Absolute values of $P_{\mathrm{he}}$ above around 0.5 indicate a significant diradical character and mean that single‐reference methods need to be applied with care.

The electron–hole correlation coefficients $R_{\mathrm{eh}}$ are presented in the bottom row of Figure 4. In line with the CASPT2 results, we consistently find positive correlation for the diradical 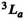 state and negative correlation for the zwitterionic 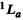 state. Again, this finding suggests that TDDFT “knows” about the diradical character of the ground state, realizing that the ground state is more similar to 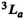 than to 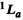. The precise $R_{\mathrm{eh}}$ values vary, highlighting that the 1TDMs produced are certainly different. Viewing these results we note, both, challenges of CASSCF/CASPT2 to describe ionic states [69] as well as challenges of TDDFT to describe exciton correlation [40] and ground‐state correlation. Thus, neither of these results is expected to reflect the true full CI solutions quantitatively, but the qualitative agreements are noteworthy.

In summary, we find that the 1TDMs computed with TDDFT reflect a number of crucial findings of the CASPT2 results. The transition dipole moments go down upon twisting. TDDFT reproduces correct signs and trends for the de‐excitations measure $P_{\mathrm{he}}$ and the correlation coefficient $R_{\mathrm{eh}}$. This analysis suggests that there is crucial physics at play and the de‐excitations are indeed a physically meaningful property of the true wave functions and certainly not simply a mathematical curiosity.

**FIGURE 5:**
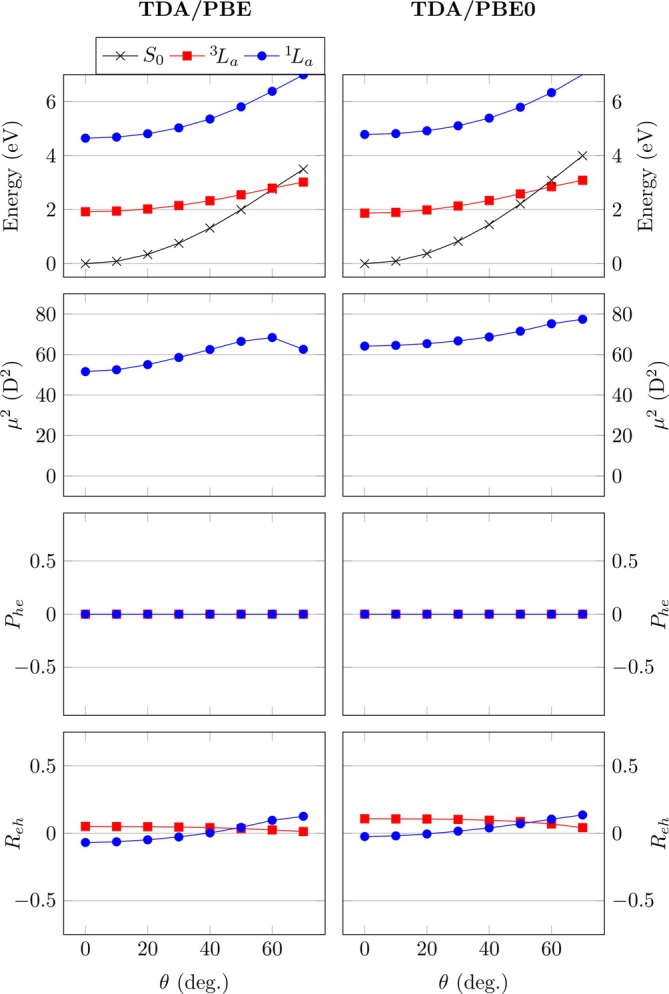
Analysis of twisting in pQDM at the TDA/PBE and TDA/PBE0 levels: Energies, squared transition dipole moments $\left(\mu^{2}\right),$ de‐excitation measures $\left(P_{\mathrm{he}}\right),$ and electron–hole correlation coefficients $\left(R_{\mathrm{eh}}\right).$

Having analyzed the full TDDFT results, it is of interest to see how much the results change if the TDA is employed and we present TDA results in Figure 5. Starting with the energies, presented in Figure 5 (top), we find that these are generally increased when using the TDA. Interestingly, the 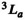 energy curves now match well with the CASPT2 ones. Converged energies for all geometries could be readily computed reflecting the higher numerical stability of the TDA. Conversely, we note that the 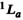 total energies are somewhat too high with the TDA, reaching about 7.0 eV at the twisted geometry, which is significantly above CASPT2.

The transition dipole moments, as shown in the second row of Figure 5, are consistently higher with the TDA than with full TDDFT. This derives from the fact that, as discussed above, the de‐excitations enter as a destructive interference term lowering the overall $\mu^{2}$ values. Crucially, the TDA even produces incorrect qualitative trends for $\mu^{2}$ where these go down with twisting for CASPT2 and TDDFT but go up with the TDA. This illustrates the point alluded to above: TDA oscillator strengths are generally on a less solid footing by not satisfying the Thomas–Reiche–Kuhn sum rule of Equation (11) and this is reflected here by the incorrect trends observed.

Proceeding to the de‐excitation measures $P_{\mathrm{he}}$, we find that these are always zero within the TDA per construction. The electron–hole coefficients ($R_{\mathrm{eh}}$) are shown in the bottom row of Figure 5. As opposed to CASPT2 and TDDFT where they curve out strongly, one finds that these are always close to zero for TDA/PBE and TDA/PBE0, with a crossing between singlet and triplet observed. One also observes a crossing between the singlet and triplet values, which is absent for the other two methods. Clearly, the $R_{\mathrm{eh}}$ values are qualitatively inconsistent between CASPT2 and the TDA.

Summarizing the TDA results, we find that all excited‐state energies go up where the triplet energies are now improved and singlet energies now overestimated. Despite some improvement in the energies, we find qualitatively incorrect physics in terms of transition dipole moments where $\mu^{2}$ values go up with increasing diradical character, whereas they should go down. Viewing the electron–hole correlation coefficients, intended to reflect diradical and ionic character, we find that also these indicate qualitatively incorrect wave function properties.

### 3.3 pQDM—Review of Energies

Following the detailed analysis of the potential curves and 1TDM properties for the different methods, we want to review the energies from a slightly different viewpoint. Table 3 presents vertical excitation energies $\Delta E_{\text{vert}}$ at the planar and twisted geometries as well as twisting energies ($E\left(70{}^{\circ}\right)-E\left(0{}^{\circ}\right)$) for the different states computed with CASPT2 and the different (TD)DFT versions. (TD)DFT values that are within 0.3 eV of the CASPT2 reference are marked in bold. Starting with the vertical 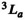 energies at the planar triplet‐optimized geometry, we find that these are notably underestimated by all DFT‐derived methods (UKS, TDDFT, and TDA) using both functionals. TDDFT/PBE0 sticks out with its vertical excitation energy underestimated by more than 1 eV, whereas the other values are within 0.5 eV of the reference. Moving to the 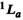 state at the planar geometry, we find excellent results (within 0.15 eV) for TDA/PBE and TDA/PBE0, whereas its energy is notably underestimated with the full TDDFT methods. At the twisted geometry, we find a slightly positive vertical excitation energy (i.e., a positive $S_{0}/T_{1}$ gap) for 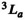 when computed with CASPT2. By contrast, this value is notably negative with UKS and TDA (and there is no converged result at all with TDDFT), highlighting that, as expected, none of these methods can provide a reliable singlet‐triplet gap near the diradical limit. As opposed to 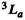, we obtain remarkably accurate vertical 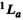 excitation energies at the twisted geometry when using TDA. Conversely, vertical 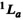 excitation energies are strongly underestimated using TDDFT.

**TABLE 3:** Comparison of vertical excitation energies ($\Delta E_{\text{vert}}$) and twisting energies (both given in eV) for the lowest excited states of pQDM evaluated at different levels of theory. (TD)DFT values within 0.3 eV of the CASPT2 reference are marked in bold.

	CASPT2	RKS/UKS		TDDFT		TDA	
		PBE	PBE0	PBE	PBE0	PBE	PBE0
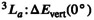	2.279	1.896	1.819	1.716	1.202	1.921	1.869
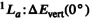	4.784	—	—	4.026	4.264	**4.647**	**4.785**
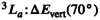	0.229	−0.593	−1.236	—	—	−0.476	−0.907
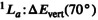	3.124	—	—	1.390	1.487	3.498	**3.007**
$S_{0}:E\left(70{}^{\circ}\right)-E\left(0{}^{\circ}\right)$	3.083	3.497	3.995	—	—	—	—
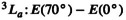	1.033	**1.008**	**0.940**	—	—	**1.100**	**1.219**
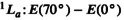	1.423	—	—	0.861	**1.218**	2.348	2.217

Having discussed vertical excitation energies we now turn to the energies required for twisting the molecule within the excited state. These energies reflect the shape of the individual potential energy surfaces, which in turn determines the outcome of geometry optimizations and dynamics simulations. Starting with *S*_0_, we find that at the CASPT2 level, an energy of about 3.1 eV is required to twist the molecule to θ=70°. Using RKS, this energy is notably overestimated (by 0.4 and 0.9 eV for PBE and PBE0, respectively). This highlights the problems of the RKS starting point. Indeed, one may wonder whether the errors in excitation energies are more of a reflection of the incorrect RKS energy than problems with TDDFT. To eliminate the influence of the RKS starting point and evaluate the behavior of the response state as such, we compute the twisting energies for 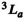 and 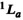. This energy for 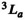 is given as about 1.0 eV at the CASPT2 level and is, indeed, very well reproduced at the UKS and TDA levels (whereas it could not be computed using TDDFT due to convergence issues). Conversely, viewing the twisting energy for 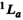, we find that this is very well reproduced by TDDFT/PBE0 whereas it is underestimated by TDDFT/PBE and strongly overestimated by TDA/PBE and TDA/PBE0.

Several considerations come into play when attempting to summarize the results from Table 3. On the one hand, we can highlight improved results for the TDA near the planar geometry, in line with general recommendations of using the TDA for standard excited‐state computations [3, 5, 6]. On the other hand, the discussion of the twisted geometry is more difficult. Consideration of vertical energies suggests that the 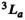 state cannot be accurately described with any method while TDA/PBE0 is the best choice for the 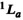 state. Conversely, consideration of the twisting energies suggests that all methods used are adequate for 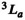 whereas TDDFT/PBE0 is the only suitable and a very reasonable choice for the 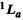 state. In summary, this suggests that a combination of UKS, TDA, and TDDFT can go quite far in the description even of systems with notable diradical character, not requiring broken‐symmetry or spin–flip approaches, but that they have to be judiciously applied for the property of interest. In particular, one could hope that total energies and potential surfaces from TDDFT computations (but not vertical energies) are potentially still useful, even when the RKS starting point is unphysically high in energy. It will be interesting to investigate similar questions for a larger set of molecules in the future.

## 4 Conclusions

It is the main thesis of this work that de‐excitations are a real physical phenomenon and that they naturally arise in quantum chemical calculations independently of the method used. Using appropriate 1TDM definitions, it is possible to provide a natural bridge between de‐excitations arising in wave function theory and TDDFT. Using the pQDM molecule as a model system, we have shown that the de‐excitation measure used here ($P_{\mathrm{he}}$) provides numerically similar results between multireference computations and TDDFT. Further interpretation of the results showed that de‐excitations do not, so much, reflect properties of the excited state but that they reflect unpaired electrons in the ground state. A more detailed analysis of electron–hole correlation suggests even that TDDFT “knows” about the diradical character of the ground state by highlighting positive correlation with the diradical 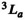 state, whereas there is negative correlation with the ionic 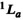 state. As a readily physically observable consequence of de‐excitations, we highlighted their effect on transition dipole moments, which, in the present case, were notably lowered through destructive interference between excitations and de‐excitations. Such alterations in transition dipole moments illustrate the well‐known more abstract statement that TDDFT fulfills the Thomas–Reiche–Kuhn sum rule regarding oscillator strengths, whereas the TDA does not [2, 3, 10].

The above results also strengthen the notion that TDDFT, as a computational method, has formal advantages over the TDA. Not only does TDDFT fulfill a number of crucial sum rules, as discussed above, but it is also able to reflect ground‐state correlation in a non‐trivial way. Clearly, such formal arguments do not mean that for any given molecule and density functional approximation, TDDFT should be better than TDA. It is not, and the TDA is certainly a powerful method in current computational chemistry. Conversely, we hope that the results presented here might reinvigorate the search for approximations that include de‐excitations in a numerically stable and beneficial way.

More speculatively, the results shown here offer some hope that singlet excited states of systems with partial diradical character can be described to reasonable accuracy with TDDFT, even in cases where the RKS reference is no longer suitable. It is too early to make concrete suggestions from the limited results presented here, but this will certainly be an exciting avenue to pursue in the future. In addition, we have highlighted that the de‐excitation measure $P_{\mathrm{he}}$ can be used as a measure of unpaired electrons in the ground state. Here, $P_{\mathrm{he}}$ stands out as a diagnostic that can be conveniently computed using standard TDDFT while it also has a rigorous basis in wave function theory.

In summary, we hope that this work will reinvigorate discussions about de‐excitations, both as a formal wave function property and regarding their practical consequences in concrete computations.

## Conflicts of Interest

The author declares no conflicts of interest.

## Data Availability

The data that support the findings of this study are openly available in Loughborough University's Repository at https://doi.org/10.17028/rd.lboro.27109336 and https://doi.org/10.17028/rd.lboro.25379311.
